# Translating Human Cancer Sequences Into Personalized Porcine Cancer Models

**DOI:** 10.3389/fonc.2019.00105

**Published:** 2019-02-25

**Authors:** Chunlong Xu, Sen Wu, Lawrence B. Schook, Kyle M. Schachtschneider

**Affiliations:** ^1^State Key Laboratory of Agrobiotechnology, College of Biological Sciences, China Agricultural University, Beijing, China; ^2^Department of Radiology, University of Illinois at Chicago, Chicago, IL, United States; ^3^Department of Animal Sciences, University of Illinois at Urbana-Champaign, Urbana, IL, United States; ^4^National Center for Supercomputing Applications, University of Illinois at Urbana-Champaign, Urbana, IL, United States; ^5^Department of Biochemistry and Molecular Genetics, University of Illinois at Chicago, Chicago, IL, United States

**Keywords:** personalized cancer models, exome sequencing, gene editing, translational research, clinical needs

## Abstract

The global incidence of cancer is rapidly rising, and despite an improved understanding of cancer molecular biology, immune landscapes, and advancements in cytotoxic, biologic, and immunologic anti-cancer therapeutics, cancer remains a leading cause of death worldwide. Cancer is caused by the accumulation of a series of gene mutations called driver mutations that confer selective growth advantages to tumor cells. As cancer therapies move toward personalized medicine, predictive modeling of the role driver mutations play in tumorigenesis and therapeutic susceptibility will become essential. The development of next-generation sequencing technology has made the evaluation of mutated genes possible in clinical practice, allowing for identification of driver mutations underlying cancer development in individual patients. This, combined with recent advances in gene editing technologies such as CRISPR-Cas9 enables development of personalized tumor models for prediction of treatment responses for mutational profiles observed clinically. Pigs represent an ideal animal model for development of personalized tumor models due to their similar size, anatomy, physiology, metabolism, immunity, and genetics compared to humans. Such models would support new initiatives in precision medicine, provide approaches to create disease site tumor models with designated spatial and temporal clinical outcomes, and create standardized tumor models analogous to human tumors to enable therapeutic studies. In this review, we discuss the process of utilizing genomic sequencing approaches, gene editing technologies, and transgenic porcine cancer models to develop clinically relevant, personalized large animal cancer models for use in co-clinical trials, ultimately improving treatment stratification and translation of novel therapeutic approaches to clinical practice.

## Introduction

The global incidence of cancer is rapidly rising, and despite an improved understanding of cancer molecular biology, immune landscapes, and advancements in cytotoxic, biologic, and immunologic anti-cancer therapeutics, cancer remains a leading cause of death worldwide. The 14.1 million new cancer cases diagnosed in 2012 are expected to dramatically increase over the next decade to 19.3 million annual cases by 2025 (1). Cancer is caused by the accumulation of a series of gene mutations called driver mutations that confer selective growth advantages to tumor cells (2). The development of next-generation sequencing technology has made the evaluation of mutated genes possible in clinical practice, allowing for identification of driver mutations underlying cancer development in individual patients. This, combined with frequency and function-based methods allows for distinguishing potential driver mutations from passenger mutations that have no effect on tumorigenesis. These advances have provided unique insights into the wide variety of genetic alterations present in an individual patient's tumor, and have spurred interest in utilizing this information to inform treatment stratification. However, translation of this genomic information into improved therapeutic approaches has not been successful for the majority of cancer patients. Therefore, as cancer therapies move toward personalized medicine, improved modeling capabilities for predicting the role driver mutations play in therapeutic susceptibility are required to address this unmet clinical need.

Recent advances in gene editing technologies such as CRISPR-Cas9 have enabled development of tumor models with specific genetic driver mutations. When applied to murine cancer models, these targeted genetic alterations have provided key insights into key mutational events promoting tumor progression and altered response to therapy (3, 4). However, many drugs showing promise in murine studies fail to translate into successful clinical trials (5), highlighting the need for improved models to better translate therapeutic efficacy, optimal dosing, and ideal combination therapies to clinical practice. Pigs represent an ideal animal model for development of genetically defined tumor models due to their similar anatomy, physiology, metabolism, immunology, genetics, and epigenetics compared to humans (6–14). In addition, their similar size permits utilization of the same instrumentation and technical maneuvers used in humans and optimized by clinicians, facilitating rapid clinical translation.

As cancer therapies move toward personalized medicine, predictive modeling of the role driver mutations play in tumorigenesis and therapeutic susceptibility will be essential. Combining porcine cancer models and gene editing technology would allow for development of clinically relevant personalized tumor models for prediction of treatment responses for mutational profiles observed clinically. Such models would support new initiatives in precision medicine, provide approaches to create disease site tumor models with designated spatial and temporal clinical outcomes, and create standardized tumor models analogous to human tumors to enable therapeutic studies. In this review, we discuss the process of utilizing genomic sequencing approaches, gene editing technologies, and transgenic porcine cancer models to develop clinically relevant, personalized large animal cancer models for use in co-clinical trials, ultimately improving treatment stratification, and translation of novel therapeutic approaches to clinical practice.

## Influence of Driver Mutations on Treatment Response

Cancer is caused by the accumulation of a series of gene mutations called driver mutations that confer selective growth advantages to tumor cells (2). The development of next-generation sequencing technology has made it possible to evaluate mutated genes in tumor cells. This, combined with frequency and function-based methods allows for distinguishing potential driver mutations from passenger mutations that have no effect on tumorigenesis. Although our understanding of the role various mutations play in driving tumorigenesis is incomplete, it is clear that genetic mutations are found in all cancers, some of which have been associated with biological characteristics of cancer (15). While all tumors result from genetic mutations, each tumor type develops mutations at different rates. In the instance of HCC, it is estimated that a single tumor contains 30–40 mutations on average, 5–8 of which are likely driver mutations (16, 17). Some of these driver mutations can have profound effects on tumor biology, having significant implications regarding diagnostics, prognostics, and therapeutic responses. For example, mutation of the tumor suppressor gene *TP53* is associated with poor prognosis and doxorubicin resistance in HCC (2, 18–20), while RAS activation is associated with resistance to sorafenib (2). Other examples include *KRAS* mutations associated with epidermal growth factor receptor antibody resistance in colorectal cancer (15), and *BRAF*^*V*600*E*^ mutations associated with positive response to vemurafenib in melanoma patients (21). As genomic analyses of clinical cancer samples continues to increase, and databases such as The Cancer Genome Atlas (TCGA) continue to grow, so does our understanding of the mutations that impact treatment recommendations. However, despite the knowledge that driver mutational profiles can have significant impacts on treatment responses, tumor genomic information is not routinely used when considering treatment strategies for the vast majority of cancer types. The lack of translation into actionable therapeutic modalities highlights the need to develop novel platforms to rapidly analyze and predict therapeutic responses for patients based on their driver mutational profiles.

## Co-clinical Trial Concept

With increased interest in testing targeted therapeutics based on driver mutational profiles in cancer patients comes a significant decrease in the number of relevant patients available for enrolment in appropriate clinical trials, significantly reducing the number of new targeted and combination therapies that can be tested. One of the new ways investigators are attempting to address this issue is through the use of co-clinical trials. Co-clinical trials are defined by the National Cancer Institute (NCI) as parallel or sequential trials of combination therapy in patients and in mouse and human-in-mouse models of appropriate genotypes to represent the patients. Utilization of mouse models that mimic the genetics of human disease in parallel to early phase human clinical trials can assist in treatment stratification by identifying patient populations most likely to benefit from treatments based on their genetic makeup. These so called “mouse hospitals” enable testing of drugs in murine models representative of multiple cancer subtypes while minimizing the cost, time, and number of human patients required (4). Co-clinical trial approaches using genetically engineered mouse models (GEMMs) have shown promise for screening therapeutics and identifying patient populations that would benefit from specific treatments (4). However, GEMMs have several drawbacks that limit the translatability of results to clinical practice. The metabolic rate of mice is substantially higher than in humans (22), and vast differences in drug metabolism and xenobiotic receptors make rodents poor models of toxicity, sensitivity, and efficacy when used in preclinical drug studies (23). The ability to establish toxicity and drug sensitivity in animal models is immensely important, as <8% of cancer drugs translate successfully from animal model testing into Phase I clinical trials (24). In addition, their small size prohibits the utilization and testing of the same tools and techniques employed in clinical practice. This is particularly important given the recent expansion of targeted locoregional ablative and arterial therapeutic strategies that reduce systemic toxicities and increase tumor drug delivery. This, combined with the fact that the genetic events required for mouse tumorigenesis differs from humans (25), highlights the need for development of improved animal models to facilitate translation of targeted and personalized therapeutic strategies to clinical practice.

### Argument for Porcine Cancer Models

Given the limitations of currently available murine and other small animal cancer models, there is a pressing need to incorporate large animal cancer models into preclinical and co-clinical therapeutic testing approaches. Pigs represent an ideal platform for development of genetically defined large animal cancer models due to their similarities with humans in size, anatomy, physiology, metabolism, genetics, epigenetics, and immunology (6–14). The life cycle of pigs also allows for development, characterization, treatment, and follow-up in a clinically relevant timeframe (26). The availability of many outbred porcine lines, high homology between the pig and human genome (27, 28), and conservation of epigenetic regulatory patterns (13) highlights the relevance of genetically defined porcine cancer models and their ability to mimic the genetic variation observed in patient populations. Pigs are also ideal models for investigation of chemotherapeutic toxicity, as the animal's basal metabolic rate and xenosensor pregnane X receptor—which is responsible for the metabolism of half of all prescriptions drugs (29)—are also very similar to humans (30, 31). Finally, their similar size allows for utilization of the same tools and techniques used in clinical practice. This is particularly important for cancers where systemic chemotherapeutic administration offers only marginal survival benefit with poor quality of life, as procedural approaches using locoregional therapeutic approaches are potentially curative therapeutic options that require further preclinical testing, but cannot be tested using similar tools in smaller animal models.

Until recently the only porcine cancer models available were spontaneous or chemically induced models (32–34). However, the sequencing of the pig genome in combination with the recent advances in targeted genome editing approaches such as CRISPR-Cas9 has allowed for development of genetically defined porcine cancer models. To date a number of genetically defined porcine cancer models capable of mimicking histological and transcriptional hallmarks of human cancer, as well as responses to cancer drug therapies have been developed. These include the Oncopig Cancer Model—a transgenic pig model that recapitulates human cancer through induced expression of heterozygous *KRAS*^*G*12*D*^ and *TP53*^*R*167*H*^ driver mutations—which has been utilized to develop HCC (35), pancreatic cancer (36), and soft-tissue sarcomas (37, 38), and a heterozygous *TP53* knockout model of spontaneous osteosarcomas (39). As genetically defined porcine cancer models continue to be developed, their use in co-clinical trial formats could provide improved prediction of patient populations that would benefit from specific treatments, improving translation of novel, targeted, and combination therapeutic strategies from preclinical murine studies to clinical practice. As our understanding of driver mutational profiles commonly observed in clinical practice continues to expand thanks to the increased use of genomic sequencing in clinical research, this information can serve as a basis for generation of additional porcine cancer models using CRISPR and somatic cell nuclear transfer (SCNT) technologies.

While there are a number of benefits associated with the use of genetically defined porcine cancer models in co-clinical trial settings, these models are not without limitation. Drawbacks of using porcine models as opposed to murine models include increased housing and husbandry requirements due to their increased size and lifespan. This limitations also limits the ability to develop, breed, and distribute multiple strains of porcine cancer models harboring different driver mutations as is currently done for murine models. In addition, specialized equipment and experience are required to ensure safe and ethical handling and use of pigs for testing experimental treatments. These animals are also raised in controlled environments that do not mimic the environmental conditions human patients are exposed to—although as this limitation is shared with murine and other cancer models, a detailed discussion of the environmental factors impacting tumor biology and treatment response is outside the scope of this review. Finally, the costs associated with development, maintenance, and utilization of porcine cancer models in co-clinical trials is significantly higher than murine models, although their use would come at a reduced cost compared to those associated with human clinical trial participants.

## Treatment Stratification Utilizing Personalized Porcine Cancer Models

**Figure 1:**
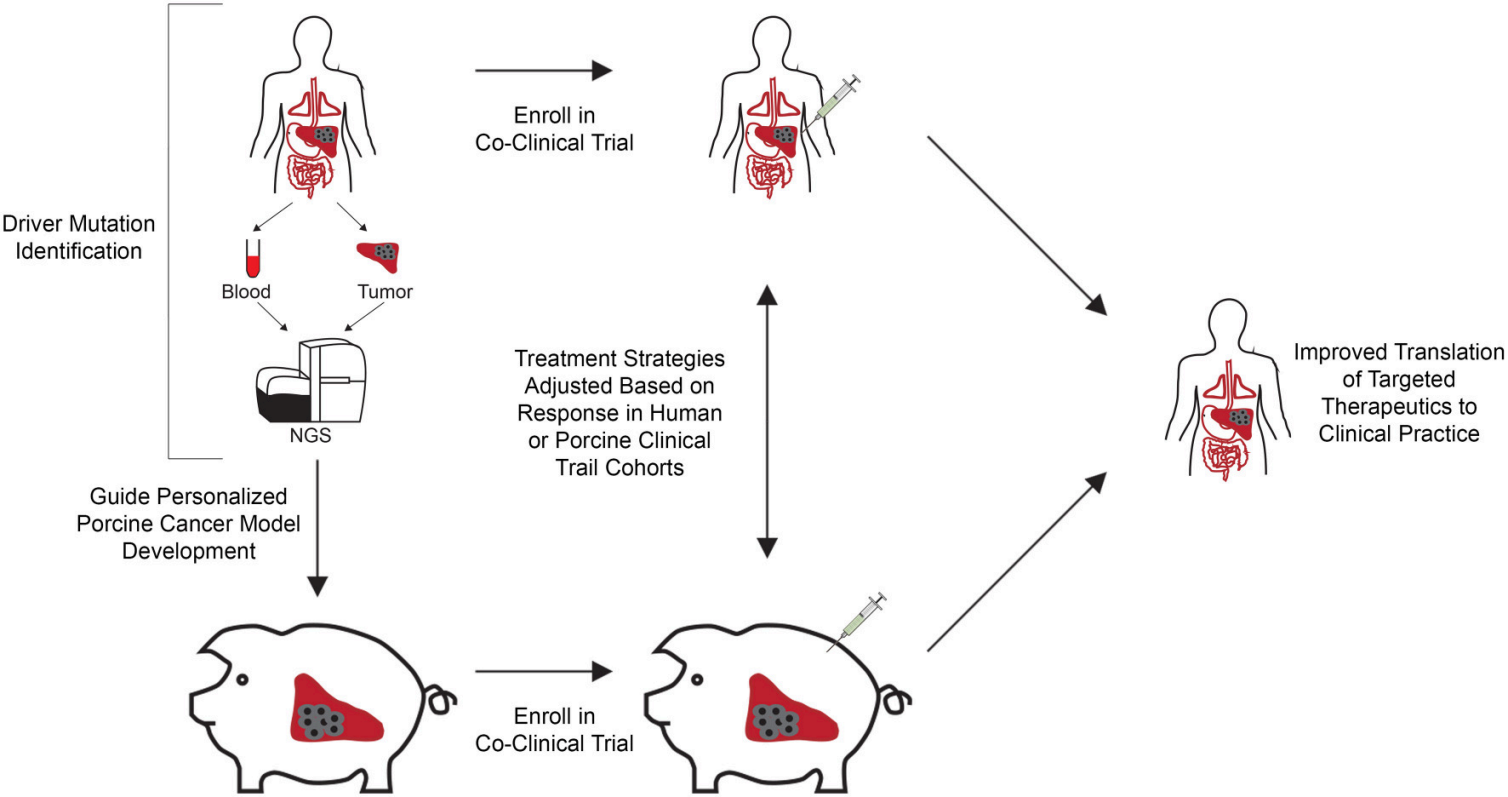
Co-clinical trial concept using genetically engineered personalized porcine cancer models. Co-clinical trials utilizing personalized porcine cancer models can improve the evaluation of cancer treatments by providing concurrent information from porcine trials on genetically relevant tumors, facilitating rapid evaluation of targeted therapeutics at reduced cost and accrual time compared to clinical trials. Patients in the clinic are screened for presence of the target driver mutational profiles and enrolled in the co-clinical trial. In parallel, porcine cells undergo CRISPR-Cas9 gene editing to develop a cohort of tumor bearing pigs harboring the target driver mutational profiles. A co-clinical trial is undertaken in which the therapeutic of interest is tested against human patients and personalized porcine cancer models harboring the same driver mutations. Therapeutic effectiveness can be rapidly evaluated in this co-clinical trial setting, reducing time and cost associated with clinical trial performance. In addition, adverse events and lack of response to therapy observed in the porcine cohort can result in early termination, reducing the costs and number of patients recruited to failed trails.

Our increased understanding of the unique genetic makeup of each patient's tumor has shed light on the fact that individual cancer varieties exist, and therefore therapies need to be optimized and adjusted to effectively treat individual patients. This optimization requires the use of preclinical cancer models representative of the driver mutational profiles of individual patients. While current co-clinical trials seek to utilize genetically defined murine cancer models in combination with human cancer patients to evaluate treatment response for patient populations, personalized porcine cancer models could transform precision medicine by providing a means to significantly improve the predictability of safety and efficacy of therapeutic drugs, devices, and procedures in co-clinical trial settings (Figure 1). Below we outline the process for developing genetically defined, personalized porcine cancer models, using the Oncopig HCC model as an example.

The first step in developing personalized porcine tumor models consists of identification of the driver mutational profile for which the treatment in question is most likely to be effective against. For targeted therapeutics, this can be done using preclinical murine models prior to proceeding with co-clinical trials utilizing personalized porcine tumor models. For repurposed compounds already approved for other cancer types, this would require knowledge of the driver mutational profiles of responding an non-responding patients. This requires performance of biopsy collection, followed by DNA extraction and genomic sequencing—for example through whole genome or whole-exome sequencing—to identify the driver mutations present. Sequencing of a control sample, such as blood, is also required to assist in distinguishing between germline and somatic mutations. Utilizing genome editing approaches such as CRISPR-Cas9, driver mutations associated with improved outcomes can be introduced into the porcine HCC cells *in vitro*. Following screening to identify cells containing the desired driver mutational profile, HCC cells are propagated for autologous injection, resulting in development of pigs bearing HCC tumors with driver mutational profiles representative of the patients of interest. Utilizing this approach, a cohort of personalized porcine cancer models can be developed in a timely fashion and utilized in co-clinical trials, significantly reducing the costs and accrual time associated with clinical trials. This approach would also provide significant benefits over murine co-clinical trials by utilizing a model animal with similar metabolism and size to humans, allowing for the same tools and techniques to be employed in both human and porcine subjects.

While the above example describes utilization of the Oncopig Cancer Model to develop personalized HCC tumors, this approach is not limited to the Oncopig and can be adjusted to facilitate development of personalized tumors for a wide variety of cancer types. However, due to the above mentioned challenges associated with developing, breeding, and disseminating multiple strains of porcine cancer models, it is unlikely that the breadth of porcine cancer models required for co-clinical trials targeting specific driver mutational profiles will ever match the number of commercially available murine models. Therefore, development of various cohorts of genetically defined porcine cancer models for co-clinical trials will likely depend on utilization of CRISPR-Cas9 to induce tumors harboring desired driver mutational profiles in individual wild type or previously produced inducible porcine cancer models. While this approach provides additional challenges compared to utilization of genetically defined murine models, it also allows for rapid development of genetically defined porcine tumor models without the extended time required to develop a pig herd harboring the desired mutational profile. In this regard, pigs harboring tumors representative of multiple driver mutational profiles could be used as their own control to confirm the effects of a given driver mutational profile on treatment response. This approach could also revolutionize personalized medicine by facilitating development of genetically unique, patient specific tumors for performance of therapeutic trials on tumors representative of the genetic profile of individual patient tumors. However, much work is still required to make this approach feasible in a timely and cost efficient manner.

### Accounting for Intratumoral Heterogeneity

One of the challenges faced when developing personalized tumor models is accounting for intratumor heterogeneity, which describes the accumulation of different genetic mutations in tumor cells within a single tumor as tumor cells evolve (40). Knowledge of the genetically diverse cell populations within a tumor can be important for guiding optimal cancer treatment decisions, and therefore the effectiveness of personalized tumor models to predict the optimal treatment strategy may be underappreciated when used to treat heterogeneous tumors. While tumor cells representative of the driver mutations modeled will be killed, the patient may develop a recurrent tumor or not respond at all due to proliferation of resistant tumor cells. These situations highlight the importance and significant challenge associated with performing clinical and co-clinical trials for targeted therapeutics, as well as the challenges of successfully employing them in clinical practice. While modeling heterogeneity represents a significant challenge for animal cancer models, the need to perform gene editing on individual pigs as described above provides an avenue through which tumor heterogeneity can be accounted for using personalized porcine cancer models. Porcine cell lines representative of multiple driver mutational profiles can be developed, mixed, and injected to develop *in vivo* intratumor heterogeneity representative of the patient population of interest. In this case, therapies will only prove effective if they're capable of eradicating all of the genetically distinct tumor cells present. Another option would consist of development of individual tumors representative of one of the genetically diverse tumor cell populations. Using this approach, treatment strategies can be applied to tumors representative of different driver mutational profiles in isolation, allowing for identification of treatment strategies most effective for each tumor cell population. However, these approaches due not take into account additional challenges associated with modeling tumor heterogeneity, including accurate identification of individual tumor clones, effects of cellular signaling and interactions between tumor cells with differential mutational profiles, and the impact of germline mutations on tumor biology.

## Conclusions

Advances in sequencing and gene editing technologies have provided significant insights into the impact of driver mutations on treatment responses for a wide range of cancer types; however, translation of this genomic information into improved therapeutic approaches has not been successful for the majority of cancer patients. We present a new personalized porcine cancer model approach leveraging clinical genomic sequence information, gene editing technologies, and transgenic porcine cancer models to develop clinically relevant, personalized large animal cancer models to better predict response to treatment in co-clinical trial settings, ultimately improving treatment stratification and translation of novel therapeutic approaches to clinical practice. Furthermore, as these techniques continue to improve, this approach could revolutionize personalized medicine by facilitating development of genetically defined porcine cancer models representative of individual patients for performance of personalized therapeutic trials.

## Author Contributions

CX, SW, LS, and KS conceptualized and wrote the manuscript.

### Conflict of Interest Statement

The authors declare that the research was conducted in the absence of any commercial or financial relationships that could be construed as a potential conflict of interest.
